# Modifications of Anti-Incontinence Procedures for Radiated Patients: A Narrative Review

**DOI:** 10.1007/s11934-026-01337-0

**Published:** 2026-04-29

**Authors:** Jonathan A. Seaman, Maia E. VanDyke

**Affiliations:** Department of Urology, University of Texas Southwestern Medical Center, Dallas, TX USA

**Keywords:** Stress urinary incontinence, Radiation, Artificial urinary sphincter, Male urethral sling, Urethral erosion, Fragile urethra

## Abstract

**Purpose of Review:**

The mainstay of surgical management for male stress urinary incontinence (SUI) for many years has been the male urethral sling and the artificial urinary sphincter. However, patients with pelvic radiation history are at increased risk for poorer outcomes. This review examines the surgical options for radiated men with SUI, how this history may affect outcomes, and surgical modifications which may optimize results.

**Recent Findings:**

Male urethral slings demonstrate diminished success but may be considered in carefully selected radiated patients. The artificial urinary sphincter remains the gold-standard for radiated men though with increased risk of complications. Key technical modifications include conservative cuff sizing, selective transcorporal placement, and consideration of lower-pressure pressure-regulating balloons. Ongoing technological innovations may address the challenges in this patient population.

**Summary:**

Successful SUI management in radiated patients requires careful patient selection, consideration of technical modifications, and thorough patient counseling regarding anticipated risks and realistic expectations.

## Introduction

Stress urinary incontinence (SUI) is prevalent in men after prostate cancer treatment, with rates of 8–23% after radical prostatectomy (RP) and 4–12% after radiation therapy (RT) [1–3]. This incidence rises to approximately 35% in those with combination therapy. Incontinence significantly impairs quality of life through social embarrassment, psychological distress, and activity limitations [1, 4]. Surgical management options range from less invasive urethral bulking agents and adjustable continence balloons to the more invasive male urethral slings and artificial urinary sphincters (AUS). Treatment choice depends on careful patient selection and counseling. The AUS remains the gold-standard for male SUI due to high patient-satisfaction and durable long-term results, even in severe cases.

Management of SUI is particularly challenging in radiated patients due to radiation-induced tissue changes [5]. Radiation causes cellular damage resulting in microangiopathy, chronic hypoxia, and chronic inflammation. Within urethral tissues, this leads to atrophy, external sphincter fibrosis, and pelvic floor sarcopenia [6]. Clinically, this decreases the efficacy of passive surgical therapies (slings, bulking agents, continence balloons) and may increase AUS complications including erosion, infection, and need for revision surgery [7–11]. These complications can be devastating, necessitating device removal, and in some cases rendering the patient ineligible for reimplantation.

Resultantly, alternative techniques and surgical modifications have been proposed to maximize success and minimize complications in this high-risk population. This review outlines surgical options for male SUI through the lens of radiation, with an emphasis on surgical considerations and technique modifications to optimize results in this challenging population.

## Treatment Modalities and Outcomes in Radiated Patients

Various treatment modalities exist for surgical management of stress incontinence. Table 1 summarizes outcomes in RT patients by procedure type.

**Table 1:** Summary of SUI surgical treatments and outcomes in radiated patients

**Surgical Option**	**Mechanism of Action**	**Success Rate in RT Patients**	**Complications in RT Patients**	**Recommendation in RT Population**
Urethral Bulking Agents	Increase outlet resistance via periurethral injection	Variable (0–83% in non-RT; worse with RT)	Frequent need for repeat intervention	Avoid entirely; contraindicated per AUA/SUFU guidelines
Male Urethral Sling	Compression and repositioning of bulbar urethra to enhance external sphincter	~ 50% success (vs. 80–90% in non-RT)	3× higher explant rate (OR 2.93); 3× higher infection rate (OR 3.06)	Consider only in strictly selected patients with mild-moderate SUI; may serve as bridge to AUS
Adjustable Continence Balloons (ProACT™)	Periurethral balloons increase outlet resistance; adjustable via scrotal ports	Poor outcomes; most studies excluded RT patients	Higher complication rates	Contraindicated per AUA/SUFU guidelines
Artificial Urinary Sphincter (AUS)	Circumferential cuff provides constant urethral coaptation	78% social continence (0–1 pads/day); 58% complete continence	Higher erosion, infection, shorter time to reoperation; higher stricture risk	Gold standard; preferred option for RT patients regardless of severity

### Urethral Bulking Agents

Urethral bulking agents increase outlet resistance by adding periurethral bulk. Despite reasonable success for female SUI, their utility in men is limited; even in non-radiated men, success rates of only 32% are reported at 6 months [12, 13]. Radiation history confers an even lower risk of success [14], presumably due to the increased fibrosis and decreased compliance of the periurethral tissues. The American Urological Association (AUA) and Society of Urodynamics, Female Pelvic Medicine & Urogenital Reconstruction (SUFU) guidelines on post-RP SUI recommend counseling patients that “efficacy is low and cure rare” and that bulking agents are best suited only for those without RT history [15]. We recommend avoiding these agents entirely in the RT population.

### Male Urethral Sling

Male urethral slings (MUS) have become popular for mild-to-moderate SUI. Tensioning of the synthetic mesh repositions the bulbar urethra, lengthening the external sphincter complex and increasing resistance. Compared to the AUS, advantages include no manual pump manipulation, no delayed activation, and no mechanical failure risk. Several variations are available globally, including adjustable and non-adjustable variations, as well as 2- and 4-arm designs. Non-adjustable slings include the Advance^TM^XP transobturator sling and Virtue^®^ 4-arm sling. Adjustable devices include the Argus, ReMeex and ATOMS (Adjustable Transobturator Male System). Significant clinical improvement may be seen in 80–90% of well-selected men: those with mild SUI (1–2 pads/day or Male Stress Incontinence Grading Scale 0–2) [16–18], intact volitional external sphincter contraction, no prior anti-incontinence surgery, low BMI, and – notably – no history of pelvic radiation [19–21].

Non-adjustable slings have been the most studied in the post-RT population, although data have been somewhat conflicting, possibly related to differences in patient selection. Some studies have failed to demonstrate a statistically significant difference in outcomes [22–24], while others have demonstrated significantly worse outcomes with initial success rates as low as 50% [25], with RT found to be an independent risk factor for failure and eventual AUS placement [21, 26]. In a 2023 meta-analysis, RT patients had lower odds of success (OR 0.68) and cure (OR 0.67) compared to non-radiated controls, were 3 times more likely to undergo sling explant (OR 2.93), and more likely to experience infection (OR 3.06) [27].

AUA/SUFU guidelines thus favor AUS over sling in the RT population, quoting a failure rate as high as 70% after sling [15]. Still, some argue that slings are viable in RT patients when strict selection criteria are met: mild incontinence, good cystoscopic sphincteric control augmented by perineal pressure, and no prior anti-incontinence surgery. In a series of 56 patients, Li Marzi et al. showed that such strictly selected patients performed similarly to non-RT patients with the AdvanceXP sling [28].

In our practice, we have adopted a similar strategy; occasionally offering MUS to highly motivated, well-selected RT patients. Because MUS does not impede future AUS placement, it remains an option, particularly for younger men who would otherwise face numerous AUS revisions in their lifetime. In this way, MUS may act as a “bridge” to AUS, delaying the need for a mechanical device with finite durability. Regardless of sling type, no surgical modifications have been described to increase sling efficacy in RT patients as placement is dependent on patient anatomy rather than tissue quality. Adjustable slings may theoretically confer greater efficacy through subsequent in-office adjustments; however, this has not been investigated in the RT population.

### Adjustable Continence Balloons

The adjustable continence balloon system (ProACT™) consists of two silicone balloons placed above the urogenital diaphragm, lateral to the urethra. Ports within the scrotum allow for in-office volume adjustments. The allure is a less-invasive alternative to MUS, but long-term outcomes are limited. In a 2023 meta-analysis, 55.2% of patients achieved 0–1 pads per day with 67% patient satisfaction [29]. There was reasonable 2- and 4-year durability but notably high heterogeneity of included studies. Importantly, most studies excluded or only included a small number of RT patients. When included, studies consistently demonstrate a lower success rate (42%) and a higher complication rate in RT patients [29–31]. Erosion requiring explant is significantly higher (23.5% vs. 4.3%) [32], thought to be related to poorly compliant post-RT tissues. As such, these devices remain discouraged in the RT population by the AUA/SUFU guidelines [15].

### Artificial Urinary Sphincter

**Fig. 1:**
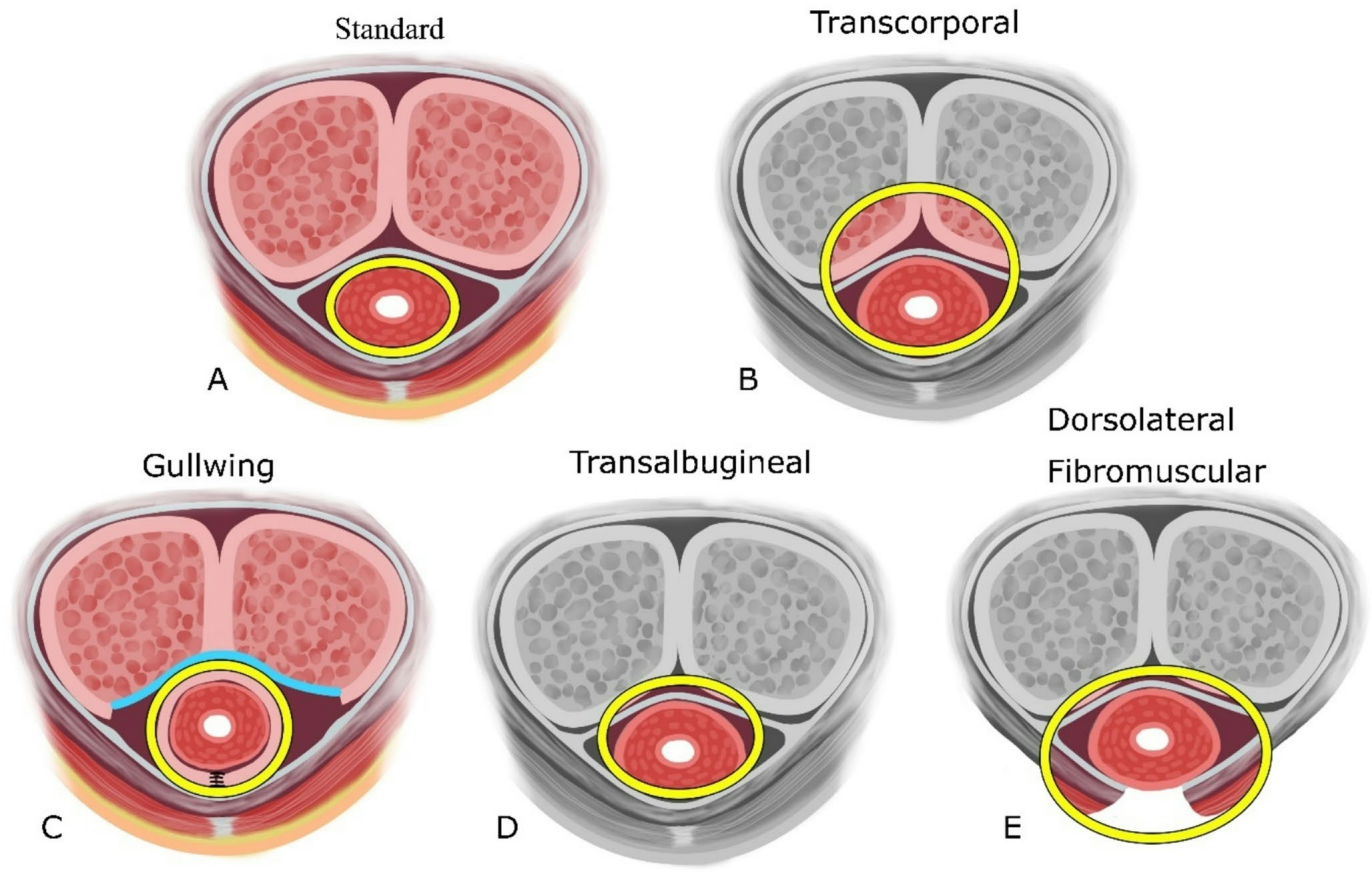
Cuff placement variations, with cuff denoted as a yellow circle. (**A**) Standard AUS placement around the bulbar urethra. (**B**) Traditional transcorporal placement, incorporating full-thickness tunica albuginea. (**C**) Gull-wing variation, with circumferential tunical wings and biosynthetic graft (blue line). (**D**) Trans-albugineal placement, incorporating the outer lamella of the tunica. (**E**) Dorsolateral fibromuscular sparing, with transection of the bulbospongiosus muscle ventrally

The AUS is the gold-standard treatment for all degrees of SUI regardless of previous prostate treatment modality. There are several devices on the global market, all of which function based on a hydraulic system and fluid-filled cuff providing circumferential coaptation of the bulbar urethra (Fig. 1A).

While the definitions of success vary, studies consistently demonstrate significant reductions in pad use and increases in quality-of-life metrics [33–35]. Success rates of 70–94% for all comers are consistently reported, with roughly 60% of patients experiencing complete continence (no pads) [36–38]. Regardless of any lingering incontinence, 90% or more patients report high satisfaction and would recommend the procedure to a friend or family member [35]. Thus, the use of the AUS is supported across all guidelines including the AUA/SUFU, European Association of Urology, International Consultation on Incontinence, Canadian Urological Association, and the Urological Society of India with strong evidence ratings [39].

In the radiated patient, efficacy is relatively preserved compared with non-radiated controls, at least in the short-term. One multi-center study found similar rates of social continence (0–1 pads/day) in radiated (78.8%) and non-radiated (78.2%) patients at 3-months with similar dry rate (58%) comparable with other published studies [7]. Other studies have suggested a higher rate of persistent incontinence after AUS in patients who underwent RT after RP compared with RP alone [8] (29.5% vs. 12.1%), although even then patient satisfaction scores appear similar.

Importantly, RT appears to increase the risk of complications after AUS. RT patients experience earlier failure and need for revision surgery, higher incidence of urethral erosion, and higher infection rates [9–11]. One study showed that RT resulted in a shorter time to all-cause device failure, including an over seven-fold increased risk for erosion [10]. Additionally, radiation increases the risk of urethral stricture [40], often necessitating device removal/revision or endoscopic procedures that may precipitate future erosion. In extreme cases of devastated outlets from multiple interventions and radiation damage, patients may require complete urinary diversion. Techniques have therefore been developed in an effort to minimize these risks and optimize AUS performance in RT patients.

## Technical Modifications for AUS Placement

Since radiation is a relative contraindication for non-AUS options, technical modifications primarily target AUS surgery. A summary of each modification and recommendation is listed in Table 2.

**Table 2:** Summary of technical modifications for AUSin radiated Pp

**Technical Modification**		**Evidence/Outcomes**	**Advantages**	**Limitations**	**Recommendation**
Conservative Cuff Sizing		Proper sizing most important factor; 3.5 cm cuff increases erosion risk dramatically	Minimizes erosion risk while maintaining continence	Some risk of persistent incontinence	When in doubt, select larger size; avoid 3.5 cm cuff entirely.
Transcorporal (TC) Placement		11% erosion vs. 28% standard (OR 0.35); 7.4% vs. 39% explant in RT patients	Lower erosion (OR 0.35) and infection (OR 0.33) vs. standard; 45% complete continence	Higher retention risk; theoretical ED risk (appears low); doesn’t protect ventral erosion. Future IPP more challenging	Safe and efficacious in select patients with atrophy or prior erosion
Gullwing Modification for TC placement	Limited to small case series; continence preserved without erosion at 2 years.		Provides circumferential urethral bulking, may improve ease of future IPP placement	Limited to small case-series, no comparative studies	Reasonable option in high-risk patients with proper counseling
Transalbugineal Modification for TC placement	76.8% 5-year social continence, 95.7% and 91.8% erosion-free survival at 12 mo and 5-years. Erectile function preserved in all preop potent patients		Avoids full-thickness corporal dissection, may protect erectile function	Limited to single case-series, no comparative studies	Reasonable option in high-risk patients with proper counseling
Rectus Fascia Wrap		4.3% erosion (1/23 RT patients); 77% complete continence	Easy harvest and placement; good continence	Single-institution experience	Conceptually sound, data limited
Dorsolateral Fibromuscular Tissue Preservation		2.9% erosion (6/208 patients, 40% RT); no RT association with erosion	Low erosion rate; may protect erectile function	Short follow-up (2.4 years); doesn’t protect ventral erosion; no head-to-head data	Reasonable option in high-risk patients with proper counseling
Lower Pressure PRB (51–60 cm H₂O)		4.4% erosion in high-risk patients; no association with RT/prior erosion	May decrease erosion risk	33% required revision for persistent incontinence	Not for universal use; adjunct maneuver for high-risk urethras

### Cuff Placement and Sizing

Early AUS surgeons universally used 4.5 cm cuffs without urethral measurement [41]. A range of cuff sizes is now available, ranging from 3.5 cm to 11 cm with most men sizing between 4.0 and 5.0 cm for virgin cases [42]. Accurate sizing is critical; the cuff must be tight enough to provide adequate coaptation, but not so tight that blood flow is compromised, potentially precipitating erosion. Cuff sizing is particularly important for the RT patient where urethral blood supply and tissue integrity may already be compromised.

Various methods have been described for measuring urethral circumference and choosing cuff size. Most surgeons utilize the sizing device provided in the AMS 800 Accessory Kit; others prefer using an umbilical tape [43]. Even among those using the sizing device, varying degrees of constriction can be applied to the spongiosum, leading to disparate measurements. We typically utilize the “push and pull” measuring technique around the most proximal straight portion of the bulbar urethra [44]. The measuring device should fit snugly without causing urethral deformation (“muffin-topping”). Some also advocate for intraoperative cystoscopy at the time of cuff placement to directly observe adequate coaptation and slight blanching of the tissue [45].

For the radiated patient, we recommend more conservative cuff sizing, as recommended by others [46, 47]. For example, when a radiated urethra measures 4.4 cm, a 5.0 cm cuff is preferred over a 4.5 cm cuff. While this may cause a higher persistent incontinence rate, the risk of erosion is theoretically minimized. Revision for cuff downsizing can always be carefully considered in those with persistent leakage.

One particular consideration is management of the narrow urethra. Introduced in 2009, the 3.5 cm AUS cuff saw early adoption, even among RT patients [44]. However, while early reports suggested similar performance compared to larger cuffs, long-term studies demonstrated increased risk of erosion and infection. This was particularly true in “high risk/compromised” urethras: those with prior radiotherapy, prior AUS erosion, or prior urethroplasty. In one series of 176 men, the majority of erosions with 3.5 cm cuffs occurred in RT patients with a 21% risk of erosion in this patient population [48]. Similarly, McGeady et al. showed an 8.6-fold increased failure risk with the 3.5 cm cuff in compromised urethras [5]. In a multi-center study of 386 patients, the 3.5 cm cuff was found to be a risk factor for erosion for all-comers, with additionally increased risk in compromised urethras [49]. As a result, 3.5 cm cuffs should be used with extreme caution in patients with urethral risk factors, including RT. We have largely abandoned their use, avoiding them entirely in RT patients and instead favoring transcorporal placement when indicated.

### Transcorporal Cuff Placement

Transcorporal (TC) placement involves incorporating a portion of corporal tissue along the dorsal urethral surface. This bolsters the urethra, increasing the diameter when robust corpus spongiosum is lacking (Fig. 1B). Although initially described by Guralnick et al. for distal cuff relocation in cases of urethral atrophy or erosion [50], indications now include any compromised urethra with high erosion risk.

A systematic review of TC outcomes in patients with compromised urethras analyzed 20 studies with 1,976 patients (849 TC vs. 1,127 standard) [51]. Pooled analysis showed 11% erosion, 7% infection, and 45% complete continence (0 pads/day). For fragile urethras (including RT), revision and explant rates were 35% and 15%, respectively. Meta-analysis of comparative studies demonstrated that TC placement had lower erosion (OR 0.35) and infection rates (OR 0.33) versus standard placement, though all studies had moderate to high risk of bias [51]. In RT patients specifically, one study reported significantly lower TC explant rates versus standard placement (7.4% vs. 39%, *p* = 0.04) [52]. TC placement notably appears to perform superiorly to 3.5 cm cuffs in the setting of urethral atrophy, with lower risk of erosion [5].

TC placement does carry a higher rate of post-operative urinary retention, presumably due to edema and increased bulk [53]. Entry into the corporal space also theoretically risks erectile dysfunction (ED) and requires corporotomy closure. This risk of long-term ED appears low, however may present challenges to future IPP placement [50]. A recent case-series reported the feasibility of IPP after TC AUS but did note difficult proximal dilation in some and a potential earlier rate of AUS cuff failure [54]. In one case report, the authors describe misplacement of the proximal IPP cylinder within the transcorporal AUS cuff and suggest direct exposure of the proximal crura to avoid this complication [55].

### Variations on Transcorporal Placement

Additional modifications to the TC technique have been described. In an effort to protect erectile function and provide additional urethral bulking, the “gullwing” modification utilizes two square-shaped flaps of ventral tunica albuginea that are wrapped circumferentially around the urethra prior to cuff sizing and placement (Fig. 1C) [56–58]. The tunical defect is then patched with a biosynthetic graft rather than primary closure as in the typical TC approach. This adds additional lateral and ventral urethral support and limits proximal corporal narrowing. However, only case reports have thus far been reported without long-term follow-up. Another technique, the transalbugineal approach, takes advantage of the bilayered structure of the tunica albuginea (Fig. 1D). Dissection is carried out under only the outer longitudinal fibers, thereby providing dorsal urethral support without entering the corpora cavernosa [59]. In their series, 12 patients with preserved preoperative erectile function remained potent after transalbugineal AUS; comparative studies to traditional TC cuff placement are lacking.

Current evidence suggests TC placement is most beneficial in atrophic or otherwise compromised urethras. In RT patients, selective use based on intraoperative findings may be appropriate, as many achieve good outcomes with standard placement when sized correctly. Our practice is to utilize TC placement more often in those with fragile urethras, particularly those with a history of prior AUS erosion, urethroplasty, or when intraoperative findings demonstrate a narrower or more atrophic appearance.

One single-surgeon retrospective study even describes placement around the entire corporal circumference and urethra [60]. Ten patients with urethral risk factors received this approach with a mean cuff size of 8.98 cm and a mean follow up of 2.5 years. Daily pad use decreased from 9.9 to 3.3, and patients were generally satisfied. Seven (70%) patients had a history of RT, and one developed ventral erosion. The utility of this approach has not been fully explored.

### Alternative Urethral Bolstering Techniques

While TC placement has been relatively well-studied, several other less-studied techniques have been described to add bulk to the urethra during AUS placement. One study describes using an autologous rectus fascia wrap between the cuff and urethra in 23 RT patients with severe urinary incontinence [47]. Over a median follow-up of 32 months, complications were described in 26%, including four patients with urinary retention (which was managed with suprapubic catheter and ultimately resolved). One patient (4.3%) with a 4.0 cm cuff presented with erosion at 3 months. Reported continence rates were notably excellent in the remaining 22 patients, with 77.3% achieving complete and 22.7% achieving social continence. The authors estimated the additional operative time to be approximately 15 min to allow graft harvest.

Another urethral wrapping technique uses porcine small intestinal submucosa (SIS) to bolster the atretic urethra. The largest such series includes 8 high-risk patients (50% prior RT, 50% prior erosion, 100% prior AUS) where porcine SIS was wrapped around the urethra anywhere from 360 to 630 degrees [61]. All patients experienced post-operative retention requiring catheterization for a mean of 14 days, and one patient required revision for wrap downsizing. At 12.4 months, 3 of 8 patients achieved complete continence, but another 3 required explant for infection and/or erosion. Notably, 80% (4/5) of those with failure had a history of radiation, while none of the successes did. The authors propose that those with prior RT may not be able to support sufficient tissue ingrowth to support AUS long-term.

Several studies report urethral buttressing through preservation of the bulbospongiosus muscle. Two of these describe the preservation of the entire muscle complex [62, 63] while another preserves the fibromuscular tissue of the perineal membrane (dorsal to the urethra) but transects the muscle ventrally, allowing direct cuff contact (Fig. 1E) [64]. Theoretically, these techniques should minimize the risk of dorsal urethral injury, buttress the urethra, and decrease erosion rates. In the fibromuscular sparing approach, 208 patients underwent AUS placement, of whom 40% had RT history [64]. At 12 months, 74% of patients with available data achieved social continence (0–1 pads per day); the probability of re-operation was 7% at one year and 17% at two years. Four patients (1.9%) developed urethral erosion and two developed device extrusion (one scrotal tubing, one scrotal pump); radiation was not found to be a contributing risk factor.

Finally, in one study of 17 patients (65% prior AUS, 53% RT history), TachoSil^®^ (a fibrin-coated collagen fleece) was placed circumferentially around the urethra prior to cuff placement in patients with intraoperative evidence of urethral atrophy. Of the included patients, 9 (53%) had radiation history. Although mean follow-up was 38 months, only 3- month continence rates were reported, with 2 patients (13%) achieving complete continence, and another 9 (56%) requiring only 1 ppd. The reoperation rate at 38 months was 25%, with one patient (6%) developing urethral erosion 13 months post-implant [65]. 

These innovative techniques are representative of the efforts to improve outcomes in patients with fragile urethras. However, they require further validation through larger comparative studies and longer follow-up; this limits the generalizability at this time. Still, they may offer an option in expert hands for well-counseled, high-risk patients.

### PRB Pressure

The pressure-regulating balloon (PRB) delivers constant circumferential pressure to the cuff while activated. Three pressure ranges are available: 51–60, 61–70, and 71–80 cm H_2_0. The 61–70 cm H_2_0 balloon is most common, although the higher-pressure balloon has been investigated for use in select patients with persistent leakage despite adequate cuff sizing [66]. Notably, this study found that all cases of erosion with the higher-pressure balloon (3/22) were in RT patients; it should thus be avoided in this population.

Alternatively, the lower pressure 51–60 cm H_2_0 balloon has been proposed to decrease erosion rates in high-risk urethras. In the largest such study, Loh Doyle et al. evaluated 90 patients deemed “high-risk,” including 75.6% with RT history, 8.9% with prior urethroplasty, and 17.8% with prior erosion or infection [67]. All patients were counseled regarding the risk of revision surgery (increase PRB rating or cuff downsizing). Post-operatively, all patients underwent delayed activation at 10–12 weeks, and routine cystoscopy to evaluate urethral integrity. At a median follow up of 46.6 months, 4 patients (4.4%) developed urethral erosion requiring device removal, with another 4 (4.4%) experiencing infection. In total, one-third of patients underwent revision for persistent incontinence at a median time to revision of 20 months, most commonly via exchange to a 61–70 cm H_2_0 PRB in over 80%. This strategy appears to lower the risk of erosion in a high-risk population, albeit at the cost of decreased overall continence rates. Notably, no erosions were seen in those patients who underwent PRB exchange to the higher-rated PRB; the authors hypothesized that the urethra may become increasingly “tolerant” of this increased pressure after a period of time with the 51–60 cm H_2_0 PRB in place.

### Nocturnal Deactivation

Given its design, the AUS provides constant circumferential urethral pressure in its “active” or “closed” state. Some have proposed teaching patients to deactivate the AUS overnight, thus providing a period of lower pressure “recovery” for the urethra. In theory, this might decrease atrophy and erosion risk and takes advantage of the fact that men with pure SUI are largely continent while supine. However, evidence for this protective effect is limited.

One study compared 60 patients who performed nighttime deactivation to 46 patients who did not [68]. In the deactivation group, 6 patients (10%) required revision surgery for urethral atrophy, and one for cuff erosion. In the non-deactivation group, 10 patients (21%) required revision for atrophy and no patient developed erosion. This difference did not reach statistical significance, although the small sample size may play a role. Importantly, this study excluded patients with a history of RT, prior AUS, or prior urethral surgery – the patients who might benefit from this technique the most. To date, no other studies have directly studied this effect. Teaching patients how to perform nightly deactivation also poses practical challenges and could potentially cause earlier mechanical failure. While an intriguing option for select high-risk patients who are dry at night, limited evidence to date supports widespread adoption.

### Other Considerations in Radiated Patients

Successful SUI surgical outcomes in RT patients require proper workup, patient selection, and counseling. This begins with a thorough history and physical examination. All SUI patients should be evaluated with cystourethroscopy to evaluate the outlet and bladder prior to SUI surgery [15]. This is particularly important for RT patients as radiation-induced strictures and radiation cystitis are prevalent and must be addressed before any SUI intervention.

In all patients, but particularly in those with a history of radiation, the clinician must differentiate between SUI and urge urinary incontinence (UUI). Bladder storage symptoms are a common side effect of pelvic radiation and can exist alongside SUI [69]. Outlet procedures will not improve – and may worsen – UUI symptoms. All patients considering SUI surgery must be counseled on the risk of persistent storage symptoms, which may require additional intervention. Urodynamic testing can be helpful to adjudicate unclear cases, as supported by the AUA/SUFU guidelines [15].

### Future Directions

Several new technologies are in development that may improve outcomes and risk profiles for RT patients. The newer Rigicon devices (the ContiClassic and ContiReflex) both feature cuffs sized at 0.25 cm increments (rather than 0.5 cm), as well as a wider range of PRB pressures, thus allowing a more customized fit [70]. In addition, the ContiReflex features an innovative “stress relief balloon” which provides additional pressure to the cuff during times of increased intra-abdominal pressure (such as a cough). Theoretically, this could allow a lower-rated PRB to be placed for baseline pressure, with the stress-relief balloon “sensing” and compensating for moments of increased abdominal pressure. Real-world performance remains to be seen.

Clinical trials are ongoing with the UroActive^®^ electronic AUS, which obviates the need for manual manipulation of a scrotal control pump. The system is instead operated by a patient remote control (PRC) or Clinician Programmer. Via the programmer, cuff pressures can be set both for baseline and lying down; these can be adjusted in the office as needed. A failsafe mechanism is deployed if the patient has not voided after 12 h, opening the cuff to allow urine egress. The first-in-human phase I study recently published 1-year results of 6 patients implanted with the device and showed promising feasibility and safety, with no patients requiring device explant at 365 days post-activation [71]. Median reduction in pad weight from baseline was 86.1% at 90 days and 86.6% at 365 days. While this device has not specifically been studied in RT patients, these technological advances allowing for individualized pressure profiles and intermittent cuff deactivation represent promising improvements over current devices.

## Conclusion

Surgical management of SUI in radiated patients requires careful patient selection, meticulous technique, and realistic expectations. The AUS remains the most effective treatment; and while numerous technique modifications are available, high-quality data and long-term follow-up are limited. Individualized surgical planning and comprehensive surgical skills allow the surgeon to adequately balance continence outcomes and complication risk. Future studies and technological innovations may further improve outcomes in this challenging population.

## Data Availability

No datasets were generated or analysed during the current study.

## Abbreviations

ATOMS: Adjustable Transobturator Male System
AUA: American Urological Association
AUS: Artificial Urinary Sphincter
ED: Erectile Dysfunction
PRB: Pressure Regulating Balloon
PRC: Patient Remote Control
RP: Radical prostatectomy
RT: Radiation Therapy
SIS: Small Intestinal Submucosa
SUI: Stress Urinary Incontinence
SUFU: Society of Urodynamics, Female Pelvic Medicine & Urogenital Reconstruction
TC: Transcorporal
UUI: Urge Urinary Incontinence
